# Applications and status of gene drive in plants

**DOI:** 10.1371/journal.pbio.3003148

**Published:** 2025-04-15

**Authors:** Bruce A. Hay

**Affiliations:** Division of Biology and Biological Engineering, California Institute of Technology, Pasadena, California, United States of America

## Abstract

Gene drive can modify or suppress plant populations, offering solutions to challenges associated with globalization and climate change. However, common features of plant biology complicate its application. Self-limiting methods provide acontrolled, reversible path forward.

Globalization—the movement of humans, plants, animals, and microbes—together with climate change, place numerous plant and animal species under novel stresses. For plants, threats include global warming, invasive species, weeds, animal pests, and vectors of disease. Harms can be mitigated or prevented through population-scale genetic alterations that introduce beneficial traits such as disease resistance or drought tolerance (population modification), or that eliminate a harmful population (population suppression). Gene drive is a possible tool for achieving these goals. DNA mediating gene drive comprises one or more genes (the drive element) that promote their own inheritance—and any linked cargo—at rates exceeding (>50%) those of other genes. This can lead to an increase in drive element frequency even if its presence results in a fitness cost to carriers. Gene drive is attractive because it is self-amplifying and self-sustaining.

One class of gene drive utilizes a Toxin-Antidote (TA) element. In nature, these often consist of two tightly linked protein-encoding genes. One encodes a toxin that is inherited by all gametes and/or progeny of a carrier; the second is an antidote that protects carriers from death. The TA element-bearing chromosome gains a relative transmission advantage by causing death of those who fail to inherit it. The first synthetic gene drive was engineered using TA logic, in *Drosophila melanogaster* in 2007 [1]. Recently, TA logic was also used to create the first synthetic gene drive elements in plants, in *Arabidopsis thaliana* [2,3]. These use a Cleave and Rescue mechanism (ClvR) adapted from earlier work in *Drosophila* [4]. ClvR uses DNA cleavage mediated by Cas9 and guide RNAs (gRNAs), followed by inaccurate repair, to create loss-of-function (LOF) alleles (the toxin) of endogenous versions of an essential gene. ClvR also includes a Rescue version of the essential gene recoded to prevent gene disruption (the antidote), which guarantees survival of carriers. In *Drosophila*, ClvR spreads because LOF alleles created in parents cause the death of progeny who lack essential gene function. In *Arabidopsis* this strategy was used to create gamete killers (Fig 1) [2,3]. A naturally occurring male gamete killer from rice, DUYAO-JIEYAO, consists of a two-gene protein–protein TA cassette [5]. Modeling suggests that gamete killers such as these can bring about population modification or suppression [2,3]. Interestingly, the DUYAO-JIEYAO element has undergone a substantial increase in frequency in Japonica rice populations in China over the last 50 years [5], demonstrating the power of such elements in nature.

**Fig 1:**
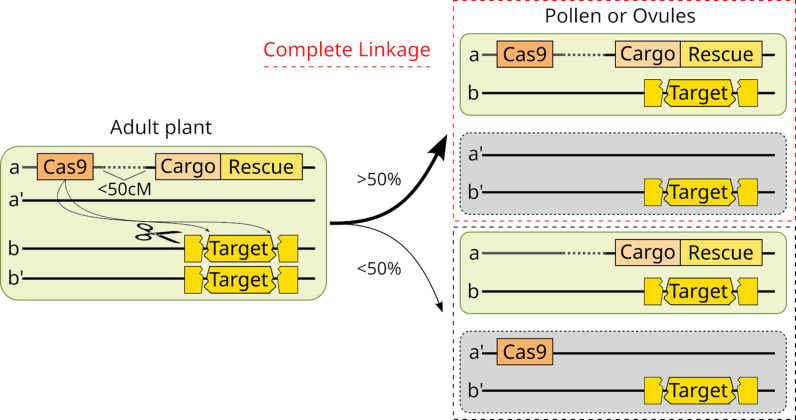
Self-sustaining and self-limiting Cleave and Rescue mechanism (ClvR)-based gamete killers. ClvR-based gamete killers produce, in the adult, loss-of-function (LOF) alleles of a gene whose expression in gametes is required for their survival. Following meiosis, these select against haploid gametes not inheriting the ClvR-bearing Rescue (gray, dying gametes), leading to almost exclusive transmission of ClvR-bearing versions of the chromosome (light green gametes) [2,3]. When Cas9 and the Cargo/Rescue are tightly linked (gamete combinations outlined in dashed red box) drive is self-sustaining because both sets of components continuously experience the drive benefits of LOF allele creation—an increase in frequency relative to that of the non-ClvR chromosome due to loss of the latter. In contrast, when Cas9 (or the gRNAs, not shown) are located at some distance from the Cargo/Rescue on the same chromosome they are initially inherited together at high frequency (thick upward arcing arrow; >50%), and drive is strong. However, over time recombination and chromosome segregation separate them into different gametes (gametes outlined in dashed black box; thin downward arcing arrow; <50%). Those carrying only Cas9 are lost, resulting in its progressive elimination from the population. Since it is the LOF alleles generated by Cas9/gRNAs that select for the presence of Cargo/Rescue by killing non-Cargo/Rescue-bearing gametes, loss of Cas9 leads to LOF allele loss and the end of Cargo/Rescue element spread. The closer these components are to each other on the same chromosome (<50% in adult plant), the longer drive is maintained—up to approximately a complete-self-sustaining element—while still being ultimately self-limited [3,6].

The above picture—that synthetic TA gene drive has great potential in many plant species—is of course subject to the constraints faced by all drive methods: that the drive components and any associated cargo are subject to mutation and selection, which can slow or prevent drive or drive outcomes [7]. Methods for maintaining TA drive element function have been addressed elsewhere [2–4,8]. Common features of plant biology, such as inbreeding (hermaphroditism and monoecy) and multiple mating partners, are also relevant to drive outcomes. These slow population modification and, particularly when inbreeding levels are high, prevent suppression [7]. Also, many species are polyploid. Polyploidy raises questions about the copy number of toxin/LOF and antidote/Rescue alleles needed to support gamete killing and survival, respectively; polyploidy also makes it challenging to identify gRNA target sites fixed in the target population. Finally, a drive element can, depending on details of the system, spread to high frequency outside the target area [7]. If hybridization intermediates are fertile, spread could also occur in other species. DNA sequence modification-based drives such as ClvR can be limited to a single species by utilizing gRNAs that recognize species-specific sites. In contrast, protein–protein-based TA systems such as DUYAO-JIEYAO, which target a conserved biological process, may support drive in other species.

The possibility of spread into non-target populations highlights a challenge of self-sustaining gene drive: that of control allowing for alteration or termination of a program (think FDA and EPA drug/chemical/product recalls as a comparison). The key to moving forward is to focus (for now) on TA gene drive that is self-limiting—drive that fades over time but that can be boosted through periodic introductions. This compromises efficiency (effort/cost) to gain control. TA-based gene drive can be made self-limiting by locating the protein toxin or Cas9/gRNAs and antidote or Rescue at some distance from each other [3,6] (Fig 1). Another system, Allele Sail, also allows for self-limiting spread of small edits at high frequency while the mendelian transgene doing the editing remains at low frequency and is ultimately lost [9].

Of course, even with self-limiting systems some level of gene flow may occur into non-target populations through simple mendelian inheritance. In this context, it is noteworthy that the majority of harvested US cropland involves GM crops [10]. Gene flow into neighboring populations does sometimes occur, yet no significant harms have been observed. This emphasizes the point that the presence of a transgene or edit (simple forms of genetic novelty) in some members of a population only constitutes an event, not an intrinsic harm.

The rigorous nature of current regulatory systems as they pertain to the introduction of genetic novelty is exemplified by the related field of classical biological control, in which a natural enemy is brought into a new environment to eat, parasitize, or otherwise suppress an invasive species. The enemy brings with it enormous amounts of genetic novelty (entire new genomes!) in the form of thousands of genes, all evolved to work seamlessly together. These genetic interventions—which are self-sustaining and not reversible—are remarkable when considered alongside self-limiting gene drive involving one or a few genes. Hundreds of successful introductions have been carried out [11]. This field demonstrates that sustained genetic interventions in wild populations can be done safely and with major ecosystem/economic benefits.

To summarize, synthetic TA-based gene drive in plants involves several challenges and requires consideration of species-specific reproductive biology and ecology. Self-limiting drive requires ongoing efforts to maintain a desired outcome. Yet given the very early stage the field is at this extra effort constitutes a feature, not a bug. Self-limiting drive embodies stewardship that values measured and reversible interventions in the face of uncertainty, while also providing a path towards use in the wild as science and regulatory policy—informed by the knowledge gained with transgenic crops and classical biological control—evolve.

## Abbreviations

**:**
ClvR: Cleave and Rescue mechanism
gRNAs: guide RNAs
LOF: loss-of-function
TA: Toxin-Antidote
